# Vitamin D boosts HIV-1 resistance in female genital epithelial cells by enhancing antiviral cathelicidin expression

**DOI:** 10.3389/fimmu.2026.1758656

**Published:** 2026-04-17

**Authors:** Sandra M. Gonzalez, Wbeimar Aguilar-Jimenez, Maria T. Rugeles, Abu Bakar Siddik, Erica Hofer Labossiere, T. Blake Ball, Ruey-Chyi Su

**Affiliations:** 1 National Sexually Transmitted Blood Borne Infection Laboratory Division, National Microbiology Laboratories, Public Health Agency of Canada, Winnipeg, MB, Canada; 2 Department of Medical Microbiology and Infectious Diseases, University of Manitoba, Winnipeg, MB, Canada; 3 Grupo Inmunovirología, Facultad de Medicina, Universidad de Antioquia (UdeA), Medellín, Colombia

**Keywords:** antiviral peptides, female genital epithelial cells, HIV-1 epithelial transmigration, HIV-1 mucosal transmission, vitamin D supplementation

## Abstract

**Introduction:**

Elevated levels of serum Vitamin D (VitD) and increased expression of its receptor on peripheral blood cells have been reported in individuals who have been exposed to HIV-1 yet remain seronegative (HESN). This study aimed to investigate the potential antiviral role of VitD in the female genital epithelium, which constitutes the first physical barrier against HIV-1. We hypothesized that VitD may modulate susceptibility to HIV-1 infection by influencing epithelial functions such as tight junction integrity, production of antiviral peptides, and secretion of pro-inflammatory mediators.

**Methods:**

Endocervical (End1), ectocervical (Ect1) and vaginal (Vk2) epithelial cell lines were cultured in transwells and treated for 24 hours with VitD (1x10^-8^M). After VitD removal, HIV-1 were added to the apical chamber, and CD4+ T cells (HIV targets) were placed in the basal chamber. HIV-1 transmigration and infection of CD4+ T cells were quantified. CAMP (cathelicidin) transcript levels and protein secretion were also measured.

**Results:**

Pre-treatment of End1, Ect1 and Vk2 epithelial monolayers with VitD reduced HIV-infection of the CD4+ T cells in the basal chamber by 50%, 53%, and 31%, respectively. Only a fraction of the HIV-1 virion applied apically transmigrated across the epithelium. The infectivity of the residual apical HIV-1 virion in the VitD-pre-treated End1, Ect1, and VK2 cultures was reduced by 42%, 36%, and 25%, respectively. These reductions were accompanied by increased RNA transcripts encoding Cathelicidin Antimicrobial Peptide (CAMP) and elevated secretion of CAMP protein into the apical chamber.

**Conclusion:**

These findings provide evidence that VitD enhances the antiviral properties of female genital epithelium and reduces HIV-1 infectivity.

## Introduction

Vitamin D (VitD), a well-known modulator of the immune activation has been shown to enhance the epithelial barrier function of enterocytes, keratinocytes and corneal cells by increasing the expression of tight junction proteins, such as claudins, occludin and Zonula occludens-1 (ZO-1) (1–4). VitD is also shown to promote the antimicrobial function of the bronchial and gingival epithelium and keratinocytes by inducing production of antimicrobial peptides such as Cathelicidin Antimicrobial Peptide (CAMP) and defensins (5–8).

The HIV-1 pandemic remains one of the most impactful and challenging infectious diseases worldwide. Globally, 40.8 million [37.0-45.6 million] people were living with HIV at the end of 2024, and still, 1.3 million [1.0-1.7 million] new infections-including 120, 000 children (<15 years old)-were reported in 2024 (9). New HIV-1 incidence rates have plateaued over the last 5 years, despite intensified efforts to increase diagnosis and treatments coverage. Young women continue to account for a substantial proportion of new infections every year (10, 11), making the female reproductive tract (FRT), one of the main entry routes for HIV-1 (12). Because not every exposure to HIV-1 results in established infection, the ability to maintain a healthy epithelium with proper epithelial integrity (13–16), may be key to preventing HIV-1 acquisition and transmission (17).

Several lines of evidence support a protective role for VitD against HIV-1 transmission (18, 19). However, whether VitD acts by reducing immune activation-thereby decreasing the number of potential HIV targets or by enhancing antiviral response at the FRT epithelium remains unclear. We previously reported a strong association between increased plasma levels of VitD and expression of its receptor (VDR) with the reduced HIV-1 infection incidence in a cohort of HIV-1 sexually exposed seronegative individuals (HESNs) (18). In support of this clinical observation, pre-treating PBMCs with VitD *in vitro* reduced cellular activation of constituent CD4^+^ T cells and consequently lowered their susceptibility to HIV-1 infection. VitD pre-treatment also increased expression of the *CAMP* gene (19). Similarly, pre-treating monocyte-derived dendritic cells (MDDCs) with VitD reduced the transfer of HIV-1 virions to CD4^+^ T cells, likely through down-regulation of HIV-capturing molecules such as DC-SIGN and Siglec-1 (20). Together, these findings suggest that multiple molecular mechanisms triggered by VitD signaling may contribute to reduced HIV-1 infection. This study aimed to define the role of VitD in preventing the establishment of HIV-infection at the FRT epithelium.

At the FRT, numerous factors-including structural differences in epithelial conformation and variations in the mucosal microenvironment between the lower and upper FRT can directly influence the outcome of exposure to HIV-1. The multilayered stratified squamous non-keratinized epithelium lining up the vagina and ectocervix at the lower FRT provides a more robust protective barrier against the virus. In contrast, the single layer of columnar epithelial cells lining the endocervix and endometrium places HIV-1 in close proximity to the target cells within the intraepithelial and sub-mucosal space (13, 17, 21). Moreover, epithelial tight junctions, overall epithelial integrity, and the production of antiviral peptides are critical in limiting viral access to the submucosa and inactivating virions (22). Consistent with this, our earlier work reported increased expression of several antiviral molecules-including cathelicidin, defensins, elafin, serpin1A, APOBEC3G, trim5α, and RNase7-in the FRT mucosa of HESNs, suggesting a role for these peptides in mediating resistance to HIV-1 (23).

This study examined and compared the effects of exposing epithelial cells derived from the endocervical, ectocervical and vaginal to the biologically active form of VitD, calcitriol, on HIV-1 infectivity using an *in vitro* mucosal model of HIV-1 exposure. Furthermore, it investigated the role of calcitriol in regulating epithelial barrier function and the production of antiviral molecules in cervico-vaginal epithelial cells.

Our findings demonstrated a reduction in HIV-1 virion infectivity following exposure to VitD-treated genital epithelial cells, accompanied by an enhanced antiviral response characterized by increased expression and apical release of cathelicidin. Although no change was detected in the RNA transcripts of genes encoding tight junction proteins, VitD treatment resulted in decreased tight junction proteins levels in endocervical and ectocervical epithelia. Collectively, these results substantiate a role for VitD/calcitriol in augmenting epithelial antiviral defenses and underscore the potential utility of this hormone as a preventive intervention against HIV-1 infection.

## Materials and methods

### Study population

Healthy donors from Winnipeg, Manitoba (Canada) were included in this study. All participants signed an informed consent approved by the Research Ethics Board at the University of Manitoba, under the project HS15280 (B2012:043, regularly renewed since 2012). Healthy donors were anonymous to the researchers involved in this study. This study was conducted according to the guidelines of the Declaration of Helsinki.

### Immortalized female genital epithelial cells and primary CD4^+^ T cells

Immortalized endocervical (End1) (ATCC CRL-2615**™**), ectocervical (Ect1) (ATCC CRL-2614**™**) and Vaginal (Vk2) (ATCC CRL-2616**™**) epithelial cells were used in this study. The cells were cultured in keratinocyte serum-free media (K-SFM) (Gibco, Amarillo, TX, USA) supplemented with bovine pituitary extract (BPE) (Gibco) at 0.05mg/mL, epidermal growth factor (EGF) (Gibco) at 0.1ng/mL and calcium chloride at 0.04 mM. Cells with lower than 15 passages were used for all the experiments. Cells were split each second day of culture using the TripLEX reagent (Thermofisher, Waltham, MA, USA) for continued growing and culturing at 37°C and 5% CO_2_ atmosphere.

Primary CD4^+^ T cells were isolated from approximately 40mL of blood from six healthy, HIV-1 negative local anonymous donors. Peripheral blood mononuclear cells (PBMCs) were obtained by a density gradient with Lymphoprep**™** (Alere Technologies AS, Oslo, Norway) and the CD4^+^ T cells were isolated as described below.

### Isolation and activation of CD4^+^T cells using magnetic beads

For CD4^+^ T cells isolation we used the negative selection Easysep**™** human CD4^+^T cell enrichment kit (Stemcell, Vancouver, BC, Canada) and the EasySep**™** immunomagnetic column-free magnet (Stemcell) according to the manufacturer**’**s instructions. Briefly, 50uL of CD4 enrichment-cocktail were added per every 2.5x10^7^ cells and incubated at room temperature (RT) for 10 minutes. Then, we added 100uL of magnetic particles per every 2.5x10^7^ cells and incubated at RT for 5 minutes. Magnets were used to enrich CD4^+^ T cells, and the purity was verified by extracellular staining with anti-CD3-PeCy5 and anti-CD4-APC antibodies (over 90% of CD4^+^ T cells). Cells were polyclonally stimulated with phytohemagglutinin (PHA) (8ug/mL) (Sigma-Aldrich) and IL-2 (50IU/mL) (Sigma-Aldrich) and cultured for 48 hours at 37**°**C and 5% CO_2_. The cells were washed before continuing the co-culture experiments to remove the PHA stimulation.

### Generation of functional epithelial monolayers

Epithelial monolayers of End1, Ect1, and Vk2 cells were generated by seeding the cells in 24-well collagen-coated transwell inserts of 6.5 mm of diameter and 0.4um pore (Corning, NY, USA), at a density of 1.5x10^5^ cells per insert and cultured for 24 hours at 37**°**C and 5% CO_2_. The optimal time to obtain a functional monolayer (measured by the epithelial integrity) was evaluated by a permeability assay with dextran-FITC (Sigma, Burlington, MA, USA) and transepithelial electric resistance (TEER). Briefly, the 70kDa dextran-FITC (Sigma) (100ng/mL) was added to the upper chamber (apical side) of the monolayers after 24, 48 or 72 hours of culture and incubated for additional 2 hours. The concentration of dextran-FITC was determined in 100uL of the lower chamber (basal side) supernatant by spectrofluorometry at 405nm wavelength. Inserts without cells were used to establish the total passage allowed by the pore size of the inserts. Permeability was calculated using the permeability coefficient, Papp (expressed in cm/second) = (dQ/dt)/(A*C0), where dQ is the accumulated Dextran-FITC in the basal compartment * basal volume, dt is the duration of the assay in seconds, A is the area of the transwell (cm^2^), and C0 is the concentration of Dextran-FITC applied at the apical side of the monolayers * apical volume (24). TEER was calculated as TEER= (Ω of samples - Ω of blank) * area of the inserts, where blank refers to the TEER from a control transwell insert without cells.

### VitD treatment of epithelial monolayers and viral transmigration assay

Functional monolayers of End1, Ect1 or Vk2 cells were treated for 24 hours at the lower chamber (basal side) with the active form of VitD, calcitriol at 1x10^-8^M [hereafter referred to as VitD] (supplemented physiological levels) resembling the ***in vivo*** uptake of VitD from circulation, or EtOH at 0.1% as vehicle control. At this point, we expected to have around 4.5x10^5^ cells per monolayer, based on the 24 hours doubling time estimated for these cells (total time in culture 48 hours). The treated monolayers were then washed with media, and the inserts were transferred into new 24-well plates containing polyclonally activated CD4^+^ T cells at the lower chamber, at a density of 3x10^5^ cells per well. The ratio for this experiment was of 1.5 epithelial cells to 1 CD4 T cell per transwell. New plates were used in order to avoid contact of the T cells with the VitD. One hundred uL of supernatants from the apical side of the epithelial monolayers were removed and 100uL of R5-tropic HIV-1 (BAL) (1 ng of p24) were added on the apical side. The co-cultures were incubated for 3 hours at 37**°**C and 5% CO_2_.

**Figure 1:**
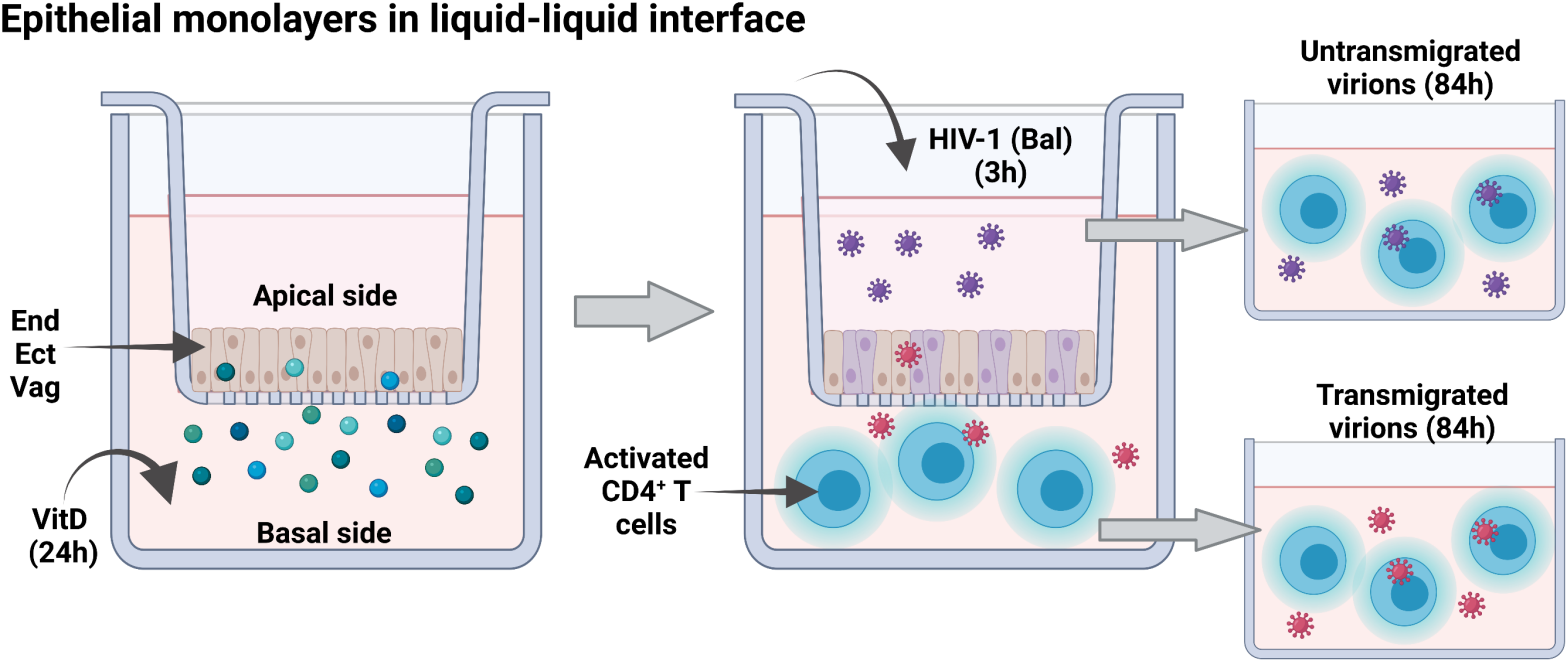
Transwell assay model. Monolayers of endocervical (End1), ectocervical (Ect1) and Vaginal (Vk2) epithelial cells grown in 24-well inserts for 24 hours in a liquid-liquid interface to obtain functional monolayers. The functional monolayers were treated with calcitriol at 1x10^-8^M or EtOH 0.1% at the basal side for 24 hours to resemble uptake of this hormone from circulation. Transmigration assays were performed for three hours, adding 1ng/mL of free-cell R5-tropic HIV-1 (Bal) to monolayers of epithelial cells at the upper chamber (Apical side). Activated CD4^+^ T cells were used as targets of infection at the lower chamber (Basal side) for evaluating infectiveness of transmigrated virions (a ratio of 1.5 epithelial cells to 1 CD4 T cells was used during the 3 hours co-culture of these cells); the remaining virions, at the upper chamber (untransmigrated), were also evaluated for their infectiveness of activated CD4^+^ T cells. The infection cultures for transmigrated and untransmigrated virions were maintained by 84 hours and infection was determined by detection of p24 by flow cytometry.

The inserts with the epithelial monolayers were then removed, and the CD4^+^ T cells with the epithelial transmigrated virions were incubated for additional 84 hours at 37**°**C and 5% CO_2_. In addition, the supernatants from the upper chamber (apical side) containing untransmigrated virions, were incubated with 3x10^5^ activated CD4^+^ T cells for 84 hours at 37**°**C and 5% CO_2_. The CD4^+^ T cells were stained for detection of intracellular p24. We performed six replicates of this assay for each epithelial cell type. The designing of the experimental model is shown in **Figure 1**).

### Detection of the HIV-1 infection of CD4^+^ T cells by flow cytometry

After incubation for 84 hours, the CD4^+^ T cells were extracellularly stained with anti-CD3-PeCy5 and anti-CD4-APC antibodies, followed by permeabilization with the Foxp3 staining kit (eBioscience, San Diego, CA, USA) and intracellular staining with anti-p24-FITC (Beckman Coulter, Brea, CA, USA). Cells were analyzed in a BD Accuri C6 flow cytometer (BD bioscience, Franklin Lakes, NJ, USA) and data analysis was performed using the FlowJo software v10. We established the region of lymphocytes, according to FSC and SSC, and determined the percentage of p24^+^CD4^+^ T cells and the Mean Fluorescence Intensity (MFI) of p24 in total CD4^+^ T cells (**Supplementary Figure 3**).

### Transcriptional expression of antiviral, tight junction and VitD pathway genes in female genital epithelial cells treated with VitD

End1, Ect1 and Vk2 monolayers were treated with VitD at 1x10^-8^M or EtOH at 0.1% during 6 or 24 hours. Cells were lysed in buffer LTR (Qiagen, Venlo, Netherlands) with β-mercaptoethanol and mRNA was obtained from lysates using the RNeasy plus Mini Kit (Qiagen), following the manufacturer instructions. The cDNA was synthetized using the SuperScript**™** III First-Strand synthesis system (Thermo Fisher Scientific, Waltham, MA USA). The transcriptional expression of nine antiviral genes, three tight junction proteins genes and three VitD pathway genes (**Supplementary Table 1**) were evaluated by SYBR green QuantiTect PCR Kit (Qiagen). All primer sets used in the study were tested for amplification efficiencies and the results were similar. Average threshold cycle (C_t_) from technical triplicate wells was determined and standardized with the 18S rRNA internal control (input control, Hs_RRN18S_1_SG QuantiTect Primer Assay, Qiagen) for relative expression level (ΔC_t_: ΔC_t (target gene)_ = C_t, (target gene)_ - C_t, (18S)_). The fold-change is calculated by normalizing or comparing the ΔCt value of the target genes of experimental group to that of the untreated (reference) group (ΔΔC_t (effects)_ = ΔC_t (target gene of treatment group)_ - ΔC_t (target gene of untreated group);_ Fold-Change = 2^-ΔΔCt (effects)^). Data is shown as fold change in RNA transcript level.

### Expression of tight junction proteins in female genital epithelial monolayers treated with VitD by confocal microscopy

End1, Ect1 and Vk2 monolayers were treated with VitD at 1x10^-8^M or EtOH at 0.1% for 24 hours. The epithelial monolayers were fixed and stained with anti-occludin-AF594 and anti-ZO-1-AF647 antibodies (Thermo Fisher Scientific). Expression of occludin and ZO-1 on the VitD- or EtOH-treated monolayers was evaluated by confocal microscope using the LSM700 (Carl Ziess, Oberkochen, Germany). Images were analyzed using the software Cell profiler (Broad Institute of MIT and Harvard) and quantification of the expression of occludin and ZO-1 is reported as Log2 of Integrated Intensity Units.

### Statistical analysis

Statistical analysis was performed using the GraphPad Prims v.9.0 software. Normality of the data was evaluated by Shapiro wilk test and comparisons between VitD and EtOH treatments were made using the Ratio paired t-test; comparisons among the three genital epithelial cell types were done by One-way Anova or Kruskal-wallis test according to normality tests. p-values lower than 0.05 were considered as statistically significant.

## Results

### Endocervical, ectocervical, and vaginal epithelial monolayers do not impede the transmigration of infectious HIV-1 virions to the basal chamber

**Figure 2:**
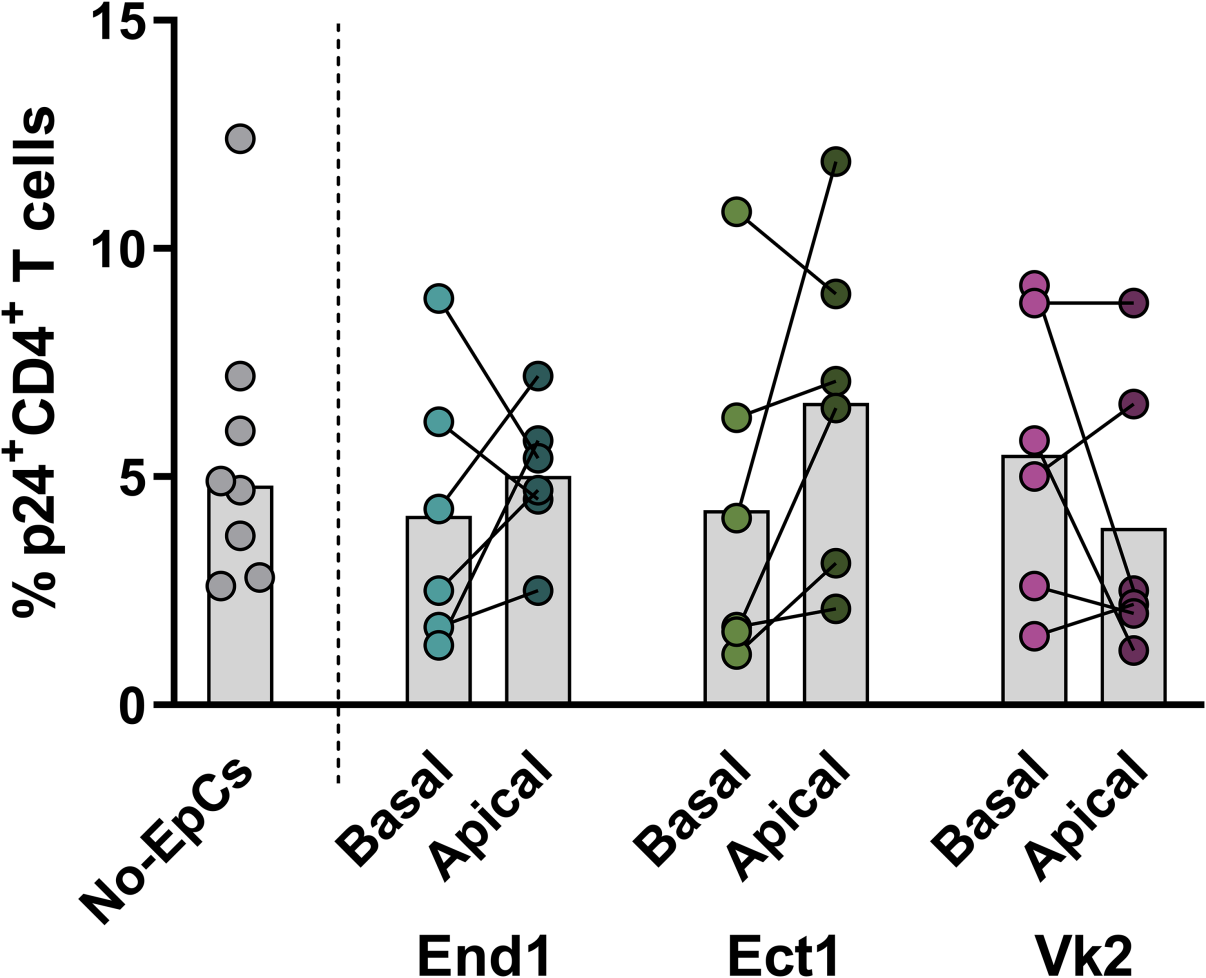
Comparison of the percentage of HIV-1 infection of CD4^+^ T cells with virions from the apical or basal sides of End1, Ect1 and Vk2 epithelial monolayers in the absence of treatment. Comparison of the frequency of infected p24^+^CD4^+^ T cells with virions from the basal side (lower chamber) and apical side (upper chamber) of transwell inserts containing untreated End1 (basal, light blue dots; apical, dark blue dots), Ect1 (basal, light green dots; apical, dark green dots) or Vk2 monolayers (basal, light pink dots; apical, dark pink dots). Infection of CD4^+^ T cells in absence of epithelial cells (No-EpCs) included as a positive control of viral infectious capacity and cell susceptibility to infection (gray dots). Comparison between infectiveness of HIV-1 virions from the apical and basal sides of each epithelial monolayer were made using the Ratio paired t-test, and comparisons among the three cell types for the HIV-1 infection with virions from the apical or basal compartments were done using One-way ANOVA. Each paired points represent one blood donor for the source of CD4 T cells, for a total of six biological replicates (i.e., 6 independent experiments).

The mucosal epithelium is critical for its barrier function in preventing pathogens entry. HIV-1, however, has been shown to be able to transmigrate through the epithelium (25, 26). Here, we assessed whether the FRT-derived epithelial monolayers ***in vitro*** could block the transmigration of infectious HIV-1 virions in the absence of treatments. Transmigration of infectious HIV-1 virions was evaluated by adding activated CD4^+^ T cells to the basal chamber, which served as targets for HIV-1 infection (**Figure 1**). In the absence of any treatment of the epithelial cells, the infectivity by the HIV-1 virions that had transmigrated to the basal chamber was compared to that of virions retained in the apical chamber (i.e., untransmigrated virions). No significant differences were observed between the basal and apical virions for End1 (basal 4.2% vs. apical 5.0%; p=0.5098), Ect1 (basal 4.3% vs. apical 6.6%; p=0.1568), or Vk2 epithelia (basal 5.5% vs. apical 3.9%; p=0.2869) (**Figure 2**). As a positive control for HIV-1 infectivity and CD4^+^ T susceptibility to HIV-1 infection, activated CD4^+^ T cells were directly exposed to HIV-1 in absence of epithelial cells (No EpCs), which yielded a similar infection frequency (5.5%; p=0.8). (**Figure 2**). These observations indicate that the presence of epithelial monolayers did not affect HIV-1 infectivity and that HIV-1 virions transmigrated through the three FRT epithelia with comparable efficiency; the epithelial monolayers did not impede viral particle transmigration.

To assess whether the three epithelial types differ in their intrinsic capacity to inactivate HIV-1 virions, we compared the infectivity of virions in the apical and basal compartments of End1, Ect1, and Vk2 monolayers. No significant differences were observed for virions in the apical compartment (End1 5.0% vs. Ect1 6.6% vs. Vk2 3.9%; p=0.2870) or for virions that had transmigrated to the basal compartment (End1 4.2% vs. Ect1 4.3% vs. Vk2 5.5%; p=0.7155). These findings indicate that End1, Ect1, and Vk2 epithelial monolayers exhibit comparable effects, if any on HIV-1 virions.

### Vitamin D–conditioned female reproductive tract epithelial cells directly attenuate HIV-1 virion infectivity

**Figure 3:**
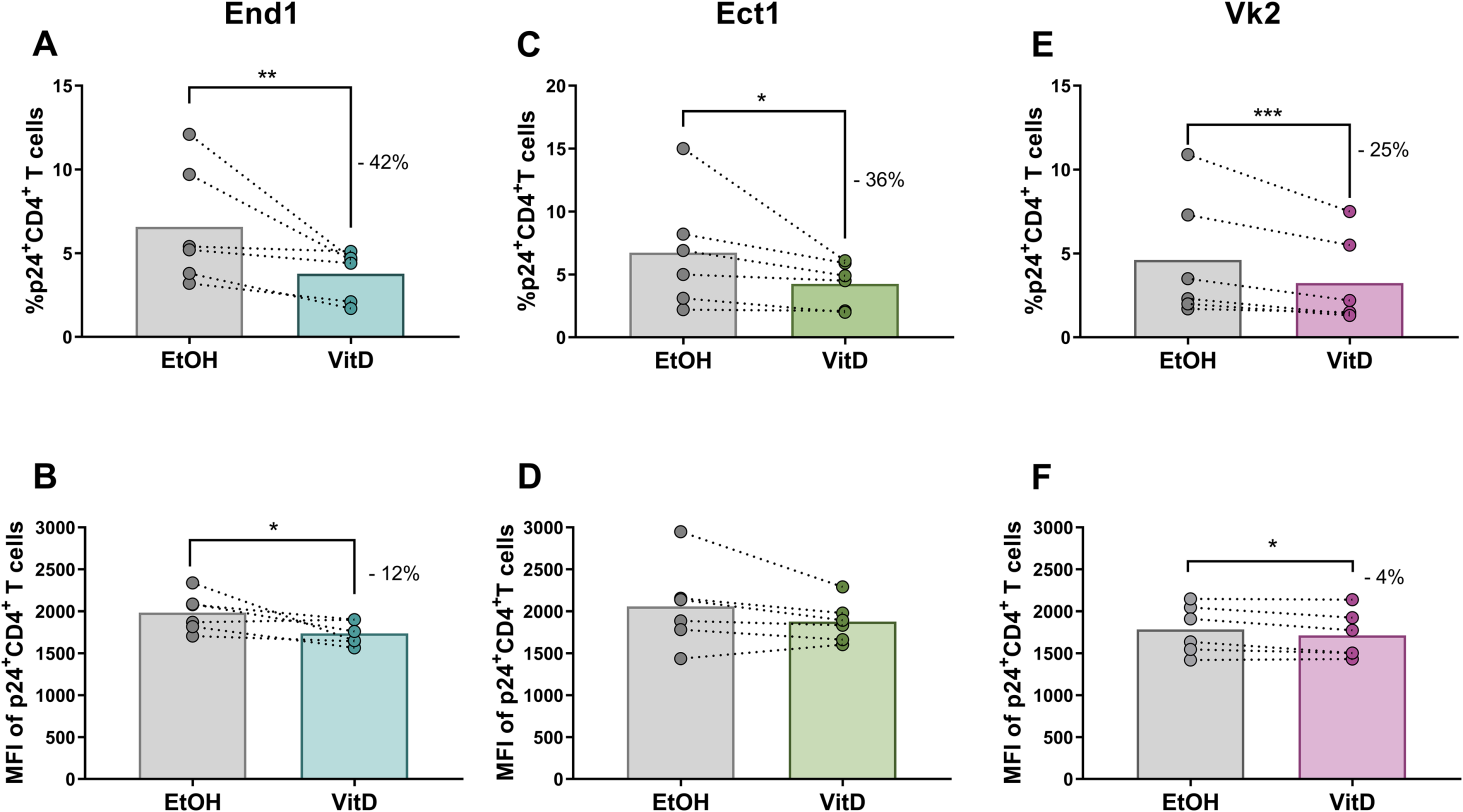
Frequency of HIV-1-infected CD4^+^ T cells and MFI of p24 in CD4^+^ T cells following the culture with virions retained at the apical side (upper chamber) of the VitD- or EtOH-conditioned genital epithelial monolayers. Comparison of the percentage of p24^+^CD4^+^ T cells following contact with untransmigrated HIV-1 Bal (1ng of p24) exposed to VitD- or EtOH-treated End1 **(A)**, Ect1 **(C)** or Vk2 monolayers **(E)**. Comparison of the MFI of expression of p24 in CD4^+^ T cells following contact of virions with calcitriol- or EtOH-treated End1 **(B)**, Ect1 **(D)** or Vk2 monolayers **(F)**. All the epithelial cells were treated with VitD or EtOH for 24 hours before co-cultures. Each paired points represent one blood donor for the source of CD4 T cells, for a total of six biological replicates (i.e., 6 independent experiments). The percentage of HIV-1 reduction by VitD compared to EtOH treatment is depicted in each figure. Comparison between treatments were made using the Ratio paired t-test, *p≤ 0.05; **p≤ 0.01; ***p≤ 0.001.

Then, we investigated whether direct contact between HIV-1 virions and VitD-treated epithelial monolayers altered viral infectious capacity. Virions were co-cultured with polarized End1, Ect1, or Vk2 monolayers that had been pre-treated with VitD at 1×10^−8^ M or with 0.9% EtOH (vehicle control) for 3 hours. To assess the infectivity of the residual HIV-1 virions remaining in the apical compartment (i.e., untransmigrated virions), these virions were subsequently transferred to cultures of activated CD4^+^ T cells. Virions exposed to VitD-pretreated End1 epithelium yielded a 42% reduction in the frequency of infected CD4^+^ T cells (mean: 6.6% vs. 3.8%; p=0.0084) (**Figure 3A**) and a 12% decrease in p24 expression relative to virions co-cultured with vehicle-conditioned End1 epithelium (MFI: 1983 vs. 1736; p=0.0276) (**Figure 3B**). Comparable reductions were observed when virions were co-cultured with VitD-conditioned Ect1 or Vk2 epithelia (**Figures 1C–F**). For VitD-conditioned Ect1 monolayers, the percentage of infected cells decreased by 36% (6.7% vs. 4.3%; p=0.0168) (**Figure 3C**), although no significant change in p24 levels was detected (MFI: 2058 vs. 1877; p=0.0922) (**Figure 3D**). For VitD-conditioned Vk2 monolayers, the frequency of infected CD4^+^ T cells declined by 25% (4.6% vs. 3.2%; p=0.0007) (**Figure 3E**), and p24 levels were also significantly reduced, though modestly, by 4% (MFI: 1784 vs. 1712; p=0.0247) (**Figure 3F**). Collectively, these findings demonstrate that exposure to calcitriol-conditioned FRT epithelial cells directly reduces HIV-1 infectivity, without affecting the expression level of HIV-1 glycoprotein, p24, in infected cells.

### Vitamin D conditioning of FRT epithelial monolayers reduced the infectivity and replication potential of transmigrated HIV-1 virions

**Figure 4:**
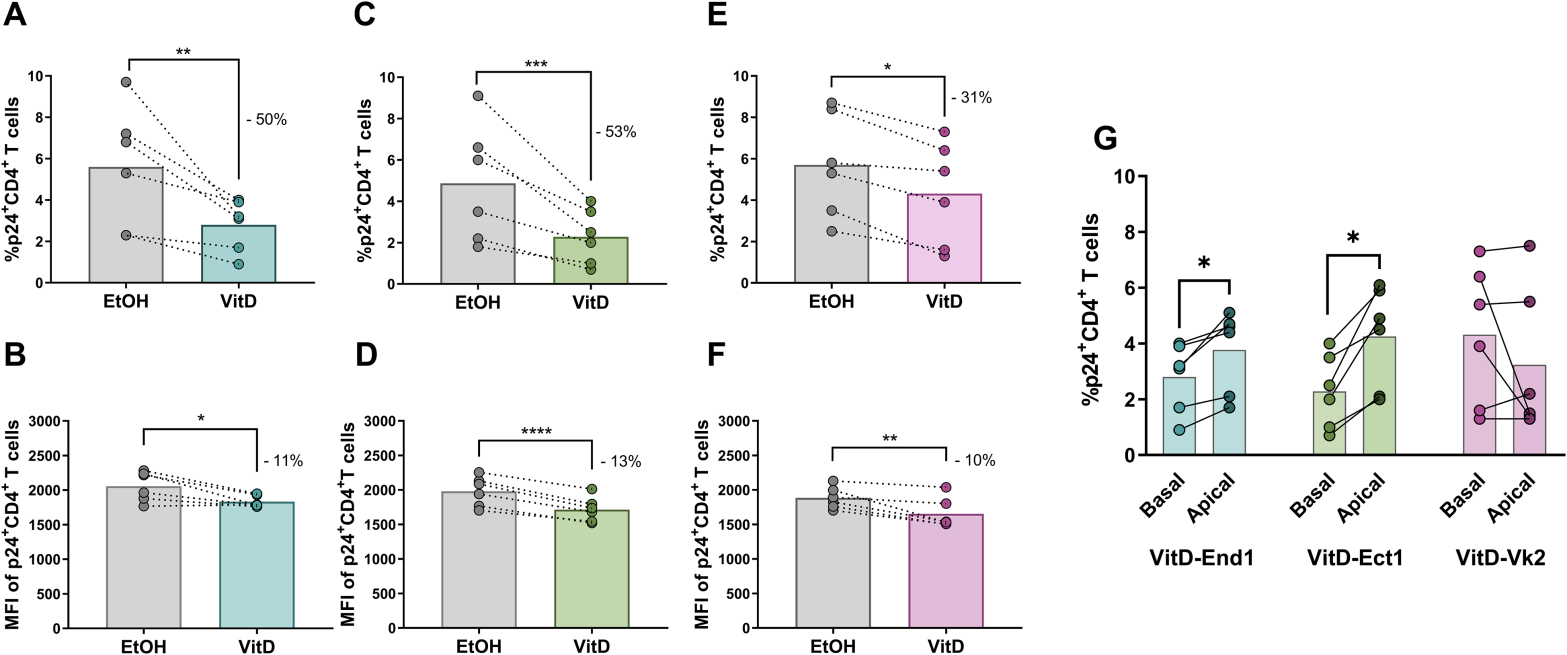
Frequency of HIV-1-infected CD4^+^ T cells and MFI of p24 in CD4^+^ T cells following the culture with virions transmigrated through the VitD- or EtOH-conditioned epithelial monolayers (lower chamber). Comparison of the percentage of p24^+^CD4^+^ T cells following culture with HIV-1 Bal (1ng of p24) transmigrated through VitD- or EtOH-treated End1 **(A)**, Ect1 **(C)** or Vk2 monolayers **(E)**. Comparison of the MFI of expression of p24 in CD4^+^ T cells following the transmigration of virions through VitD- or EtOH-treated End1 **(B)**, Ect1 **(D)** or Vk2 monolayers **(F)**. Comparison of the frequency of p24^+^CD4^+^ T cells infected with virions from the basal side (lower chamber) and apical side (upper chamber) of VitD-treated End1(light blue dots represent the basal compartment; dark blue dots represent the apical compartment), Ect1 (light green dots, basal; and dark green dots, apical) or Vk2 (light pink dots, basal; and dark pink dots, apical) monolayers **(G)**. All the epithelial cells were treated with VitD or EtOH for 24 hours before co-cultures. A ratio of 1.5 epithelial cells to 1 CD4 T cells was used during the 3 hours co-culture of these cells. Each paired points represent one blood donor for the source of CD4 T cells, for a total of six biological replicates (i.e., 6 independent experiments). The percentage of HIV-1 reduction by VitD compared to EtOH treatment is depicted in each figure. Comparison between VitD and EtOH treatments and comparison between infectious virions at the apical and basal sides of each epithelial monolayer were made using the Ratio paired t-test, *p≤ 0.05; **p≤ 0.01; ***p≤ 0.001; ****p<0.0001.

To determine whether transmigration through VitD-conditioned End1, Ect1, and Vk2 epithelial monolayers reduced the infectivity of HIV-1 virions, we added activated CD4^+^ T cells to the basal chamber of VitD- or EtOH-pretreated epithelia. The infectivity of virions was reduced after transmigration through VitD-conditioned End1, Ect1, and Vk2 monolayers compared with EtOH-conditioned controls (**Figures 4A–F**). HIV-1 virions that transmigrated through VitD-conditioned End1 epithelium exhibited a 50% reduction in the frequency of p24^+^ CD4^+^ T cells (mean: 5.6% vs. 2.8%; p=0.0021) (**Figure 4A**) and an 11% decrease in p24 expression per cell (MFI: 2058 vs. 1832; p=0.0103) (**Figure 4B**). Likewise, virions transmigrated through VitD-conditioned Ect1 monolayers yielded a 53% reduction in the frequency of infected CD4^+^ T cells (4.9% vs. 2.3%; p=0.0007) (**Figure 4C**) along with a 13% decrease in p24 levels in infected cells (MFI: 1981 vs. 1716; p<0.0001) (**Figure 4D**). For virions transmigrated through VitD-conditioned Vk2 monolayers, the frequency of infected CD4^+^ T cells was reduced by 31% (5.7% vs. 4.3%; p=0.0183) (**Figure 4E**), and p24 expression decreased by 10% (MFI: 1846 vs. 1654; p=0.0052) (**Figure 4F**), relative to EtOH-conditioned Vk2 epithelia. Taken together, these findings indicated that transmigration through calcitriol-pretreated FRT epithelial monolayers reduced HIV-1 infectivity—evidenced by lower frequencies of infected CD4^+^ T cells—and impaired viral replication, as reflected by diminished p24 expression.

**Figure 5:**
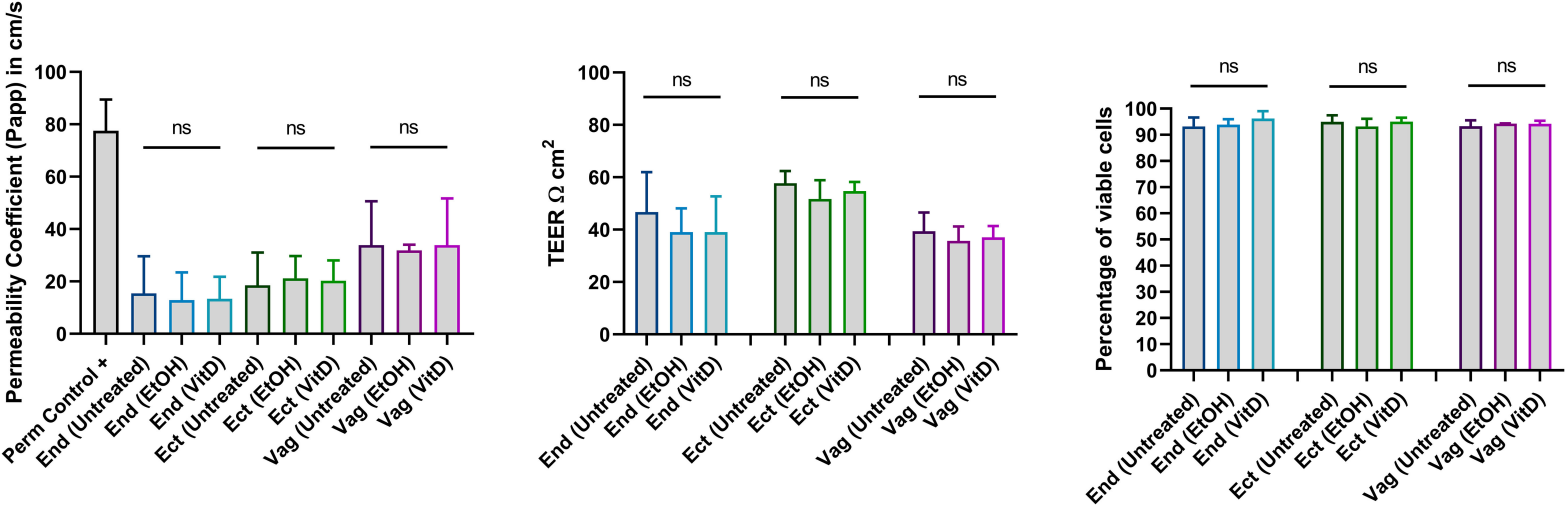
Epithelial integrity and viability of the genital epithelia monolayers after VitD treatment. The integrity of the epithelial monolayers was evaluated by permeability of the Dextran-FITC and transepithelial resistance (TEER), following 24 hours of treatment with EtOH or VitD or no treatment (Untreated). TEER= (Ω _samples_ - Ω _blank_) area of the transwell insert (0.6 cm^2^), where blank refers to the TEER from a control transwell insert without cells. Treatment with Triton-X 1% was included as a positive control (Perm control+) of monolayer leakage in Dextran-FITC diffusion assay. The viability of the genital epithelial cells was evaluated by trypan blue staining at 24 hours post-treatment. Three independent experimental replicates (3 transwell filters per experimental condition, per cell line examined) were shown in the figure. Conditions were compared by Friedman test. ns, no significant.

We next assessed whether VitD-conditioned End1, Ect1, and Vk2 epithelia exhibited enhanced inactivation of transmigrated HIV-1 virions compared with virions retained in the apical compartment. The rate of infection in the basal chamber was therefore compared with that of virions remaining apically. Notably, HIV-1 virions that transmigrated through VitD-conditioned End1 and Ect1 monolayers showed a further reduction in infectious capacity relative to their apical counterparts (End1: basal 2.8% vs. apical 3.8%; p=0.0313; Ect1: basal 2.3% vs. apical 4.3%; p=0.0313). This additional reduction was not observed for VitD-conditioned Vk2 monolayers (basal 4.3% vs. apical 3.2%; p=0.8125) (**Figure 4G**). These findings support that transmigration through VitD-conditioned FRT epithelial monolayers further diminished HIV-1 infectivity. Importantly, treatment with either VitD or EtOH did not affect epithelial cell viability (End1 p=0.3611; Ect1 p=0.1944; Vk2 p=0.5278) or the monolayer integrity, assessed by transepithelial resistance (TEER) (End1 p=0.2222; Ect1 p=0.1944; Vk2 p=0.3611) and dextran-FITC diffusion assays (p=0.9444; Ect1 p>0.9999; Vk2 p>0.9999) (**Figure 5**).

### VitD increased expression of VitD pathway hydroxylase CYP24A1 in End1, Ect1, and VK2 monolayers

**Figure 6:**
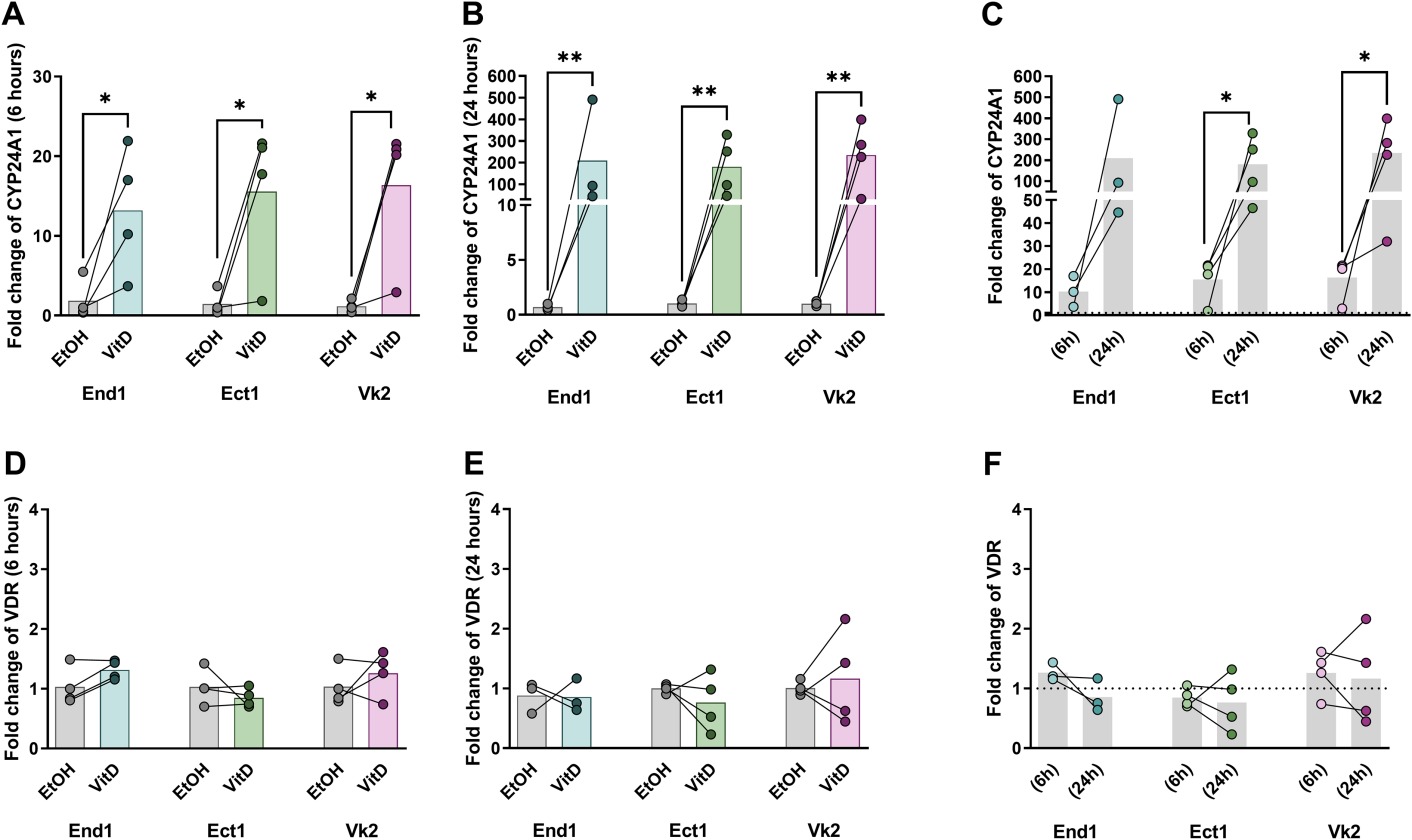
Transcriptional expression of VitD pathway genes in genital epithelial cells after VitD treatment. Fold changes of expression of CYP24A1 at 6h **(A)** and 24 h **(B)** of VitD or EtOH treatments of End1, Ect1 and Vk2 cells, and comparison between 6h and 24h time points **(C)**. Fold changes of expression of VDR at 6h **(D)** and 24 h **(E)** of VitD treatment of End1, Ect1 and Vk2, and comparison between 6h and 24h time points **(F)**. Fold-change in transcript (2^-ΔΔCt (effects)^) was calculated using ΔΔC_t(effects)_ = ΔC_t(target gene of treatment group)_ - ΔC_t (target gene of untreated group)_. Average threshold cycle (C_t_) of 3 technical PCR replicates of the target gene was standardized against the average C_t_ of the 18S rRNA (internal input reference) of the corresponding sample, termed ΔC_t (target gene)_. Shown was the data of 4 independent experiments (i.e., 4 transwell filters per treatment group, per cell line). Ratio paired t test was used. *p≤ 0.05; **p≤ 0.01.

To confirm the epithelial response to VitD treatment, we evaluated the transcriptional expression of genes involved in the VitD pathway. As expected, expression of the hydroxylase *CYP24A1*—which is upregulated in response to elevated VitD levels to promote its degradation^27^—was markedly increased at 6 hours by 7.2-fold in End1 (p=0.0198), 10.8-fold in Ect1 (p=0.0243), and 14.1-fold in Vk2 cells (p=0.0118) relative to EtOH-conditioned controls (**Figure 6A**). After 24 hours of VitD treatment, *CYP24A1* expression further increased by 296-fold in End1 (p=0.0058), 176-fold in Ect1 (p=0.0015), and 232-fold in Vk2 monolayers (p=0.0010) (**Figure 6B**). A significant rise in expression from 6 to 24 hours was observed in Ect1 (p=0.0371) and Vk2 (p=0.0337) monolayers, with a similar trend detected in End1 cells (p=0.0676) (**Figure 6C**), supporting a sustained activation of the VitD pathway in**/**VitD/calcitriol-conditioned genital epithelia. In contrast, expression of VDR remained unchanged at both time points following VitD treatment (**Figures 6D–F**).

We also investigated the molecular mechanisms by which VitD affects mucosal epithelial cells of the FRT—and considering previous reports describing the hormone**’**s role in tight junction (TJ) regulation (1–4)—we examined changes in the expression of occludin and ZO-1 in VitD-conditioned End1, Ect1, and Vk2 monolayers. VitD treatment did not alter the RNA transcript levels of occludin or ZO-1 at either 6 or 24 hours compared with EtOH-conditioned controls (**Supplementary Figures 1A–D**). Because TJ proteins are essential components of interepithelial junctions, contributing to selective barrier formation and maintenance of cell polarity, we further evaluated TJ protein abundance and localization by confocal microscopy. In contrast to the transcript data, confocal imaging revealed a reduction in protein expression intensity 24 hours after VitD treatment in both End1 monolayers (occludin: p<0.0001; ZO-1 p<0.0001) (**Supplementary Figures 1E, F, G**) and Ect1 monolayers (occludin: p<0.0001; ZO-1: p<0.0002) (**Supplementary Figures 1E, H, I**). The basal occludin and ZO-1 expression in Vk2 monolayers was generally lower than that in End1 or Ect1 cells (**Supplementary Figure 1E**), and no significant differences in protein levels were detected following VitD treatment (occludin p=0.7745; ZO-1 p=0.8710) (**Supplementary Figures 1E, J, K**) Together, VitD did not alter the expression of TJ genes but selectively reduced occludin and ZO-1 protein levels in End1 and Ect1, without affecting their localization or expression in vaginal tissue-derived Vk2 epithelial monolayers.

### VitD treatment induces cathelicidin expression in End1, Ect1, and Vk2 monolayers with apical extracellular release

**Figure 7:**
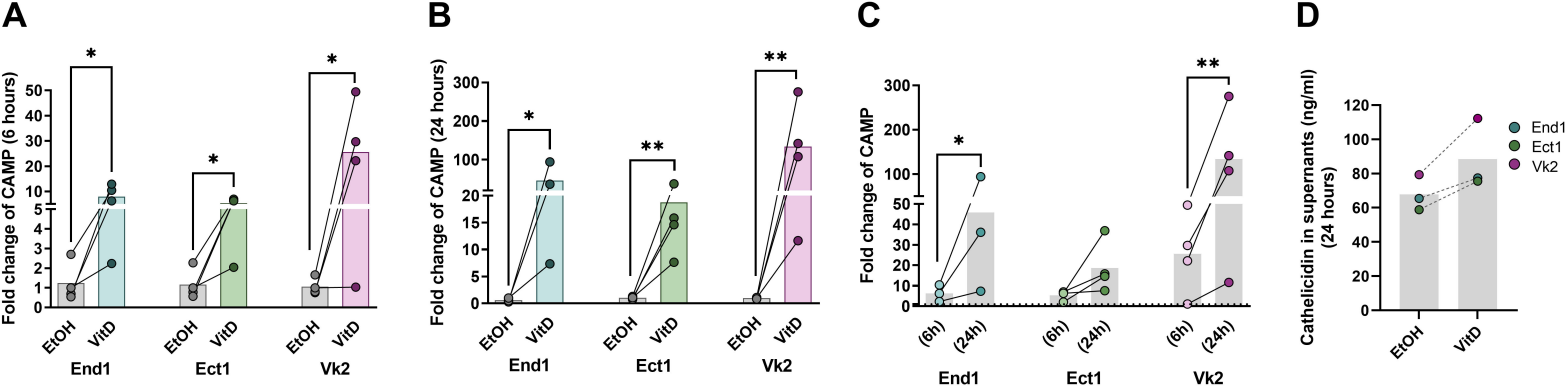
Expression of cathelicidin (CAMP) gene and protein in genital epithelial cells following VitD treatment. Fold changes of the CAMP RNA transcripts at 6h **(A)** and 24 h **(B)** following VitD- or EtOH- treatment of End1, Ect1 and Vk2, and comparison between 6h and 24h time points **(C)**. Fold-change in transcript (2^-ΔΔCt (effects)^) was calculated using ΔΔC_t(effects)_= ΔC_t(target gene of treatment group)_ - ΔC_t (target gene of untreated group)_, as described in **Figure 6**. Shown was the data of 4 independent experiments (i.e., 4 transwell filters per treatment group, per cell line). Ratio paired t tests were used. *p≤ 0.05; **p≤ 0.01. The amount of cathelicidin protein released into the apical side of End1, Ect1 and Vk2 monolayers was assessed using ELISA **(D)**. Three independent experiments (3 transwell inserts) for each cell type were performed. The supernatant from the apical side of the monolayers were pooled and concentrated using speed vacuum; concentrations shown were adjusted according to concentration factor, to nanogram of cathelicidin per ml of supernatant.

Given that the VitD-induced antiviral peptide cathelicidin has been shown to inactivate HIV-1^28^, we examined the transcriptional expression of several antiviral genes—*CAMP*, *TRIM5α*, *ELAFIN*, *SLPI*, *SerpinA1*, *APOBEC3G*, and *SAMHD1*—after 6 and 24 hours of VitD treatment (**Figure 7**; **Supplementary Figure 2**). The *CAMP* transcript, which encodes cathelicidin, increased at 6 hours by 6.4-fold in End1 cells (p=0.0180), 4.7-fold in Ect1 cells (p=0.0143), and 24.3-fold in Vk2 cells (p=0.0282) compared with EtOH-conditioned monolayers (**Figure 7A**). Further elevations were detected at 24 hours, with *CAMP* expression rising by 51.3-fold in End1 (p=0.0338), 18.4-fold in Ect1 (p=0.0013), and 133.2-fold in Vk2 monolayers (p=0.0038) (**Figure 7B**). These time-dependent increases were significant for End1 (p=0.0277) and Vk2 (p=0.0044) and showed a trend for Ect1 (p=0.0642). In contrast, VitD treatment did not affect the expression of *TRIM5α*, *ELAFIN*, *SLPI*, *SerpinA1*, *APOBEC3G*, or *SAMHD1* at either 6 or 24 hours (**Supplementary Figure 2**). In summary, VitD selectively induced robust upregulation of the cathelicidin-encoding *CAMP* gene across all three epithelial monolayers, while exerting no detectable modulatory effects on the expression of other antiviral restriction factors.

To determine whether the transcriptional upregulation of the *CAMP* gene translated into increased peptide production, we quantified cathelicidin protein levels in apical and basal supernatants of End1, Ect1, and Vk2 monolayer cultures after 24 hours of VitD treatment using ELISA (**Figure 7D**). Elevated cathelicidin concentrations were detected in the apical compartment of VitD-conditioned End1 (77.4 ng/ml), Ect1 (75.5 ng/ml), and Vk2 (112.3 ng/ml) monolayers compared with their respective EtOH-treated controls (65.5 ng/ml, 58.8 ng/ml, and 79.4 ng/ml) (**Figure 7D**). Cathelicidin was undetectable in the basal supernatants of all VitD-treated monolayers (data not shown). These findings demonstrate that VitD stimulation enhanced cathelicidin production and promoted its preferential release toward the outward-facing apical surface of the genital epithelium. Together, these results indicate that VitD drives a sustained cathelicidin response in genital epithelial monolayers, with apical secretion that may contribute to diminishing the infectious capacity of HIV-1 virions upon direct contact with calcitriol-treated epithelia.

## Discussion

The female genital epithelium is the primary barrier against heterosexual HIV-1 transmission, but the virus can breach this defense through abrasions or viral transmigration (27, 28). Factors such as epithelial structure, antiviral peptides, microbiota, hormonal fluctuations, and mucosal immunity (24, 28–30), can influence transmission. Vitamin D (VitD), a secosteroid hormone with immunomodulatory effects, is converted to calcitriol, which regulates immune responses (31–33). Previous work has shown that VitD reduces HIV-1 susceptibility in CD4^+^ T cells and enhances antiviral peptides (20), and that higher VitD levels in HESNs correlate with stronger antiviral responses and reduced immune activation (19).

This is the first study, as far as we are aware, to investigate the direct effects of VitD on the female genital epithelium and its impact on HIV-1 mucosal transmission through the FRT. Our experimental approach, using an *in vitro* monolayer model of mucosal exposure (**Figure 1**) facilitated the study of the direct effects of VitD on cellular properties of genital epithelial cells with minimum confounding factors, such as structure of the epithelium. That is, the shedding, and cornification of the epithelial cells may dilute out the effect of VitD and may impede the transmigration of viral particle to hinder the proper assessment of VitD effects. This monolayer culture condition facilitated the observation of the intrinsic response of epithelial cells to VitD and their subsequent effect on the infectious capacity of virions in the absence of other protective mechanisms via the mechanical and structural features of the different epithelia. To be noted is a potential caveat of this monolayer model that it could not address the effect of VitD on the cornification, the translocation of adherens junction protein, and the shedding of the vaginal and ectocervical epithelial cells (42).

With this genital epithelial monolayer model, we found that VitD treatment significantly reduced HIV-1 infectivity in End1, Ect1, and Vk2 epithelial monolayers, with lower frequencies of infected CD4+ T cells and reduced p24 expression per infected cell (**Figure 3**). Furthermore, transmigrating virions through VitD-treated cells showed decreased infectivity (**Figure 4**), suggesting that VitD directly impacts HIV-1 virion capacity. Importantly, VitD did not alter epithelial integrity (**Figure 5**), indicating that these effects are independent of changes in the physical barrier. Although we could not quantify the number of translocated virions due to assay limitations, these findings highlight a novel role for VitD in modulating HIV-1 infectivity at the female genital epithelium. Although VitD has been reported to affect the tight junctions of gut epithelia (1–4), this monolayer model is not sufficient to examine any synergistic effects that VitD may have on the conformation of the multilayer squamous vaginal or ectocervical epithelia. Such study requires optimization of culture conditions, allowing maximum exposure/penetration of VitD and HIV-1 viral particles through the multilayers, organoids or tissue culture.

Currently, no comparative studies evaluate differences in viral epithelial transmigration across these three FRT epithelial monolayers. However, differences in viral inactivation capacity have been described in other mucosae. In adult and fetal oral and fetal intestinal epithelia, where *in vitro* transmigration was observed, only adult epithelial cells were able to inactivate viral particles via an antiviral response mechanism (23, 26). In contrast, polarized tonsil, cervical, and foreskin epithelial cells internalized dual-tropic HIV-1 virions into vesicular compartments during transmigration, where they remained infectious for several days (27). Although virions were detected in the basal chamber of the epithelial cultures, most were retained in intracellular vesicles (27). Similarly, in VK2 cells, rapid viral internalization was observed, followed by a steady reduction in viral infectivity as early as the viral sequestering phase (23). On the other hand, primary vaginal epithelial monolayers demonstrated reduced viral internalization, with increased retention of infectious virions extracellularly attached to the apical side during transmigration assays (34). Further studies are needed to explore the differential viral transmigration and inactivation capacities of epithelial cells across the FRT.

Although we compared the infectious capacity of virions remaining at the apical side of End1, Ect1, and Vk2 monolayers with those transmigrated to the basal side, we observed no significant differences in viral inactivation between the two compartments. These findings suggest that previously reported differences in the *in vivo* susceptibility of these epithelia in the FRT (22, 28, 30) may be more related to structural factors, such as conformation of the epithelium, thickness, and microenvironment, rather than intrinsic differences in viral inactivation capacity. For example, the endocervical mucosa, composed of a single columnar epithelial layer, may be more susceptible to HIV-1 transmission compared to the multilayered ectocervical and vaginal epithelia (35, 36). However, the larger surface area and heightened immune activation in the vagina and ectocervix likely make these sites the primary entry points for HIV-1 into the submucosa (28, 30). Future studies comparing the infection risk across these anatomical regions and evaluating monolayer versus multilayer models of ectocervical and vaginal cells will be important. While our findings provide valuable insights, it is important to note a few considerations for translating these results to *in vivo* HIV-1 transmission: i) we used immortalized genital epithelial cells rather than primary cells, and ii) the monolayers were differentiated at a liquid-liquid interface rather than an air-liquid interface. These factors may influence the cellular phenotype, differentiation state, and immune activation levels in the model. Interestingly, when comparing virions from the apical and basal compartments of VitD-treated End1 and Ect1 monolayers, there were further decreases in infection for virions that had transmigrated through these epithelial layers, compared to untransmigrated virions (**Figure 4G**). This suggests that VitD may enhance inactivation mechanisms associated with transmigration. Whether HIV-1 transmigrates via transcytosis (30) in our model and whether intracellular mechanisms induced by VitD are contributing to these observations warrants further investigation.

The reduced infectivity of HIV-1 observed following exposure to VitD-treated End1, Ect1, and Vk2 monolayers may be mediated by increased production of the antiviral peptide cathelicidin, as we observed a significant upregulation of this gene in all the epithelial monolayers (**Figure 7**). The decreased infection at both the apical and basal sides of the transwell assays supports the hypothesis that VitD enhances viral inactivation, either through direct exposure to the treated epithelial monolayers or by the release of antiviral factors from these cells. Further studies investigating viral interaction with conditioned media in the absence of epithelial cells could offer additional insights into this protective mechanism. Cathelicidin has been shown to prevent HIV infection through multiple mechanisms, including targeting and permeabilizing the lipid membrane, inhibiting early steps in the HIV replication cycle, and blocking HIV reverse transcriptase (27). These mechanisms may also explain the observed reduction in p24 level in the infected cells that were exposed to VitD. In line with our findings, VitD-induced upregulation of antiviral molecules has been reported in other epithelial cells, including keratinocytes, and oral and lung epithelial cells, and is associated with improved responses to various infections (5–7, 37). However, an *in vivo* study on oral VitD supplementation found no significant changes in cathelicidin levels in cervicovaginal fluids (CVF) following treatment. This discrepancy may be due to the route of VitD administration, with localized supplementation in the FRT potentially offering greater benefits. Indeed, while systemic VitD levels were elevated after supplementation, the study also found a decrease in VitD levels in CVF (28). The possibility that additional antiviral molecules, produced by epithelial cells in response to VitD treatment and not evaluated in our study, may also contribute to our findings cannot be excluded.

Although the level of RNA transcripts of the tight junction protein genes was not affected by VitD, VitD significantly reduced the expression of both occludin and ZO-1 protein in End1 and Ect1 monolayers, albeit a very modest amount (**Supplementary Figure 1**). This reduction could be mediated by intracellular degradation of these proteins induced by VitD, or through a blockade of transcript translation. This mechanism, as well as any effects of VitD in the location of TJs and formation of cell-cell junctions warrants further investigation. Moreover, further investigation on the effects of VitD on adherens junction proteins, which were not evaluated in this study, but are preferentially formed in vaginal and ectocervical squamous epithelia, should also be considered. Again, no changes in epithelial integrity were observed in the monolayers (**Figure 5**).

The physiological significance of reduced tight junction protein levels in preventing viral infection remains unclear, though subtle effects may have been overlooked due to the limitations of the study model. Contrasting reports have shown that calcitriol enhances the *in vitro* expression of claudin-2 and -12 in enterocytes via the VitD receptor, contributing to calcium absorption (1). VitD has also been shown to induce occludin and claudin-14 expression in bladder epithelial cells, thereby enhancing the epithelial barrier during urinary tract infections (38). Additionally, VitD helps preserve intestinal barrier integrity by reducing permeability and altering the fecal microbiome in mice (39). In summary, while VitD treatment reduced the expression of tight junction proteins occludin and ZO-1 in epithelial monolayers, it did not affect epithelial integrity, and the implications of these changes for viral infection prevention remain unclear, warranting further investigation into potential underlying mechanisms.

## Conclusion

Our findings suggest that VitD plays a protective role in reducing HIV-1 transmission during mucosal exposure, likely through its induction of the antiviral peptide cathelicidin in female genital epithelial cells. This effect warrants further investigation *in vivo*, particularly through direct VitD supplementation at the female genital tract (FGT). Notably, we have previously reported reduced CD4+ T cell susceptibility to HIV-1 infection following calcitriol treatment (20, 40), and a recent study showed that VitD oral supplementation increases epithelial thickness in the cervical mucosa while decreasing CD4 expression in the lamina propria and epithelium, potentially reducing HIV-1 gp120 binding (28). Taken together, these findings underscore the potential of VitD as a modulator of HIV-1 mucosal transmission, with implications for localized therapeutic strategies at the FGT.

## Data Availability

The original contributions presented in the study are included in the article/supplementary material. Further inquiries can be directed to the corresponding author.
