# Supplementary material for: Vitamin D boosts HIV-1 resistance in female genital epithelial cells by enhancing antiviral cathelicidin expression
Source: Front Immunol. 2026 Apr 17;17:1758656. doi: 10.3389/fimmu.2026.1758656 (PMC13132785; doi:10.3389/fimmu.2026.1758656)
Supplement: Supplementary file 1 [file DataSheet1.docx]

Supplementary Material

## Supplementary Figures

**
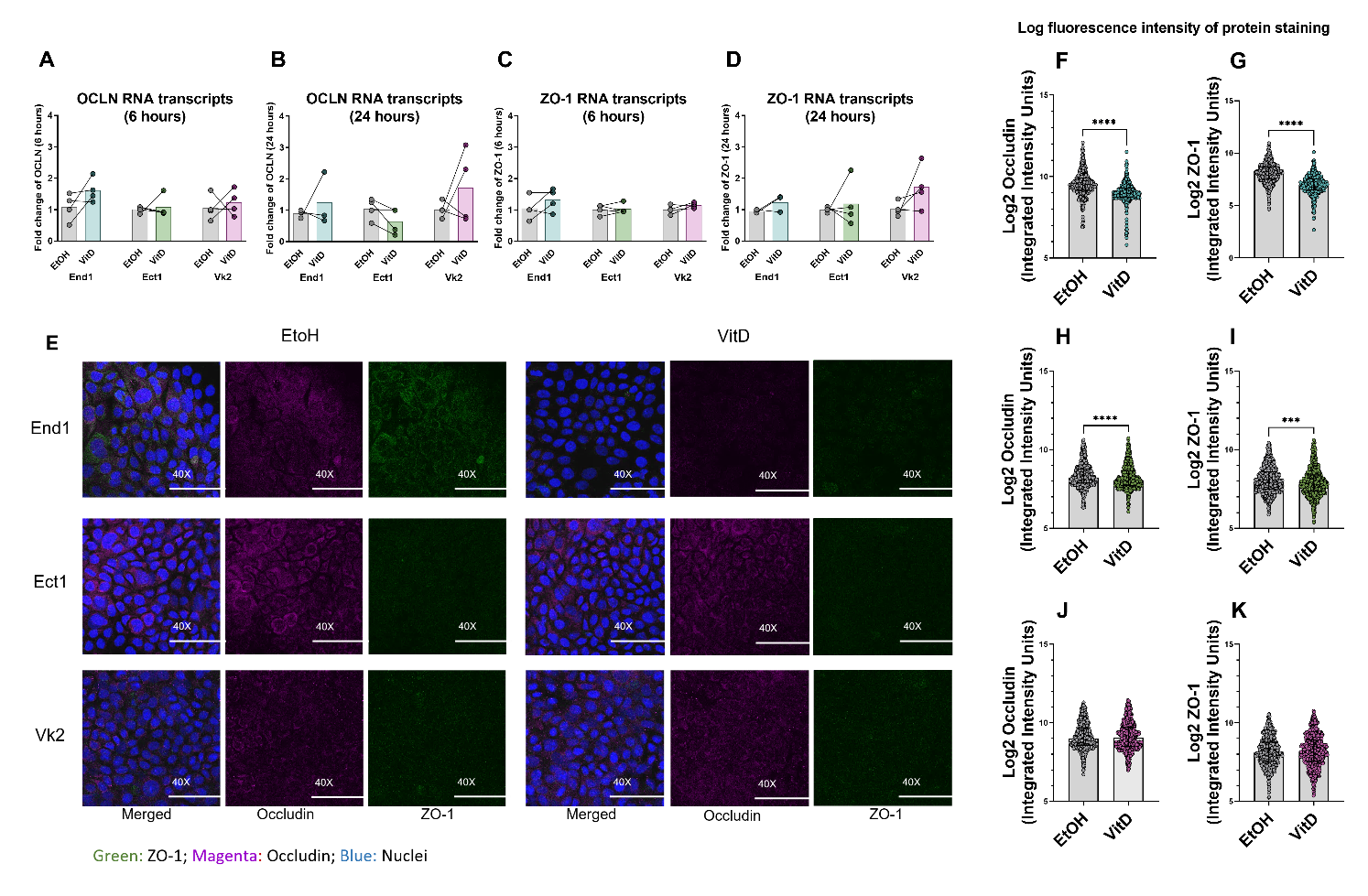
Supplementary Figure 1.**

**Supplementary Figure 1. Expression level of tight junction genes and proteins in genital epithelial cells following VitD treatment.** Fold changes of RNA transcript level of Occludin and ZO-1 genes at 6h (A) (C) and 24 h (B) (D) of VitD- or EtOH- treated End1, Ect1 and Vk2 cells. Fold-changs in transcript level was calculated using standard formula (ΔΔC_t (effects)_ = ΔC_t (target gene of treatment group)_ - ΔC_t (target gene of untreated group);_ Fold-Change = 2^-ΔΔCt (effects)^). Average threshold cycle (C_t_) of 3 technical PCR replicates of the target gene was standardized against the average C_t_ of the 18S rRNA (internal input reference) of the corresponding sample, termed ΔC_t (target gene)_. Shown was the data of 4 independent experiments (4 transwell filters per treatment group, per cell line). Comparison between EtOH and VitD treatments were done using ratio paired t test. (*) p≤ 0.05. Protein level of occludin and ZO-1 on End1, Ect1 and Vk2 monolayers after 24 hours of VitD treatment by confocal microscopy (E). Merged image on the left, occludin staining on the middle (red), ZO-1 staining on the right (green) of each treatment. Quantification of the fluorescent intensity of occludin and ZO-1 protein in End1 (F) (G), Ect1 (H) (I) and Vk2 (J) (K) were performed using cell profiler, Integrity density transformed to Log2 and reported as Log2 Integrated Intensity Units. Each dot corresponds to the Integrated Intensity Units in one cell, and cells in eight randomly selected fields of each transwell filter were included per each condition. Comparison between EtOH and VitD treatments were done using ratio paired t test. (*) p≤ 0.05; (**) p≤ 0.01(***) p≤ 0.001; (****) p<0.0001.

**Supplementary Figure 2.**


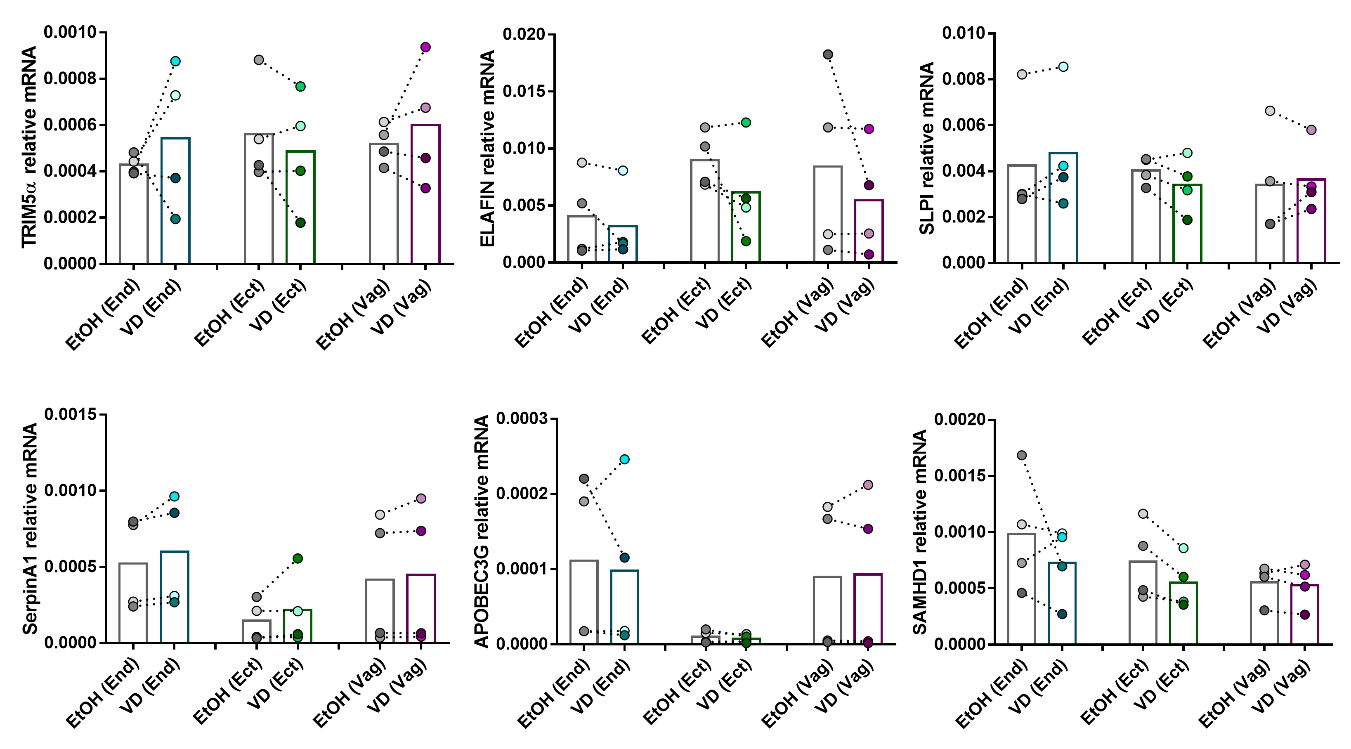


**Supplementary Figure 2. Transcriptional expression of antiviral genes in genital epithelial cells after VitD treatment.** Relative expression of TRIM5α, ELAFIN, SLPI, SerpinA1, APOBEC3G and SAMHD1 in End1, Ect1 and Vk2 cells following 6, 12, 24 and 48 hours of stimulation with VitD (1x10^-8^M) or EtOH (0.1%). Lightest color corresponds to 6h and darkest color to 48h. Transcriptional expression relative to housekeeping gene 18s. Comparison between EtOH and VitD treatments were done using ratio paired t test. Four technical replicates included per cell type and time point.

**Supplementary Figure 3.
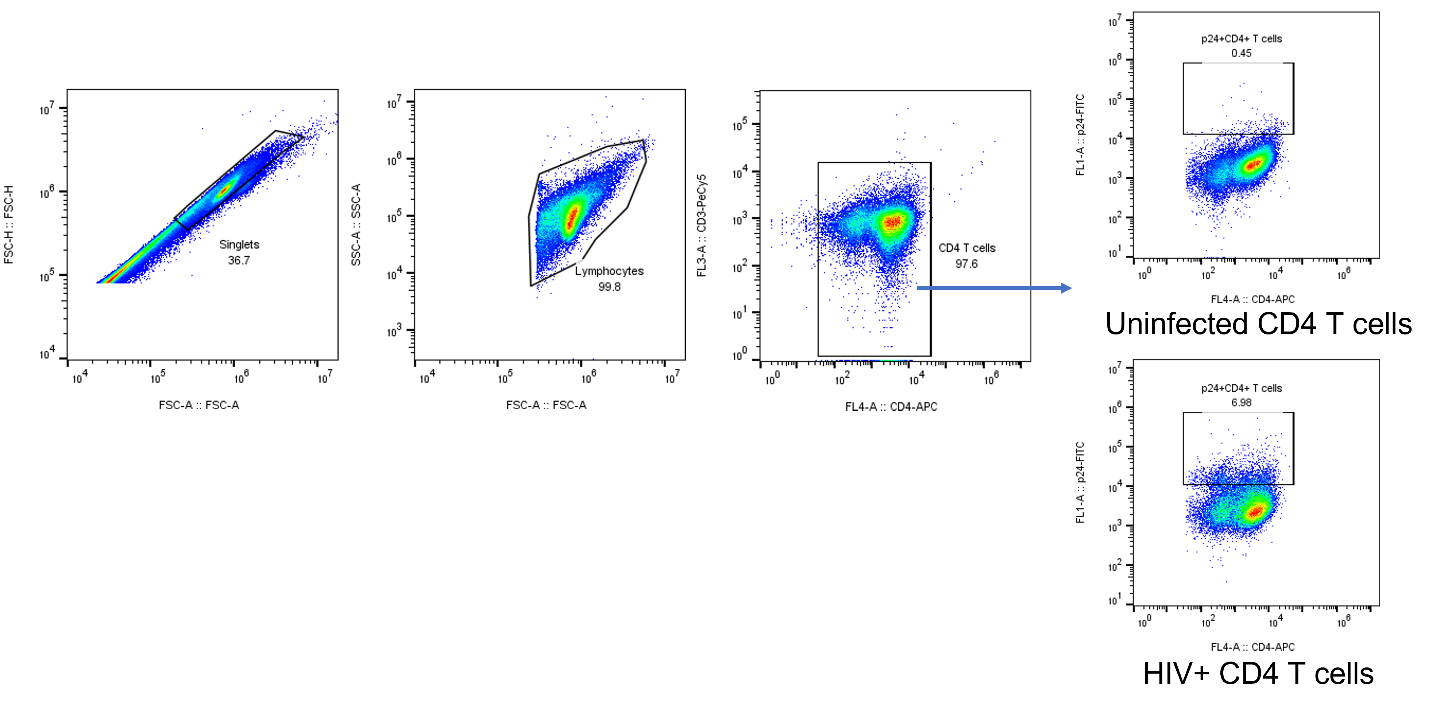
**

**Supplementary Figure 3. Gating strategy to define HIV-infected CD4 T cells.** Data acquisition was performed in the BD AccuriC6 flow cytometer and data analysis was done in the FlowJo Software v10. The aggregates and debris were excluded and the region of lymphocytes was established. The cells co-expressing CD3 (PeCy5) and CD4 (APC) were selected from the lymphocytes gate to evaluate the cells positives for p24 (FITC) (HIV+). A control of the unspecific binding of the anti-p24-FITC antibody was included with staining of uninfected CD4 T cells.

## Supplementary Tables

**Supplementary Table 1.** Primer sequences for the antiviral response, tight junctions and Vitamin D pathway genes.

| Gene | Primers |
| --- | --- |
| CAMP | Fw: 5’-GGATGCTAACCTCTACCGC-3’  Rv: 5’-AGGGTCACTGTCCCCATACA-3’ |
| ELAFINA | Fw: 5’ AAACACCTTCCTGACACCATGA 3’  Rv: 5’ TTAACAGGAACTCCCGTGACAG 3’ |
| APOBEC3G | Fw: 5’-CCGTCTGGCTGTGCTACGAA-3’  Rv: 5’-GCTTCCTCCACTTGCTGAACCA-3’ |
| SLPI | FW 5’-GATGTTGTCCTGACACTTGTGG-3’  RV 5’-CTTTCACAGGGGAAACGCAGG-3’ |
| HBD2 | Fw: 5’-GCTTGATGTCCTCCCCAGACT-3’  Rv: 5’-CCTATACCACCAAAAACACCTGGA-3’ |
| HBD3 | Fw: 5’-GCCTGTTCCAGGTCATGGAGG-3’  Rv: 5’-TCGGCAGCATTTTCGGCCAG-3’ |
| TRIM5 | Fw: 5’-TTCTGTCAGGAGGACGGGAA-3’  Rv: 5’-GCTTCTGCCTCAGCATCTC-3’ |
| SAMHD1 | Fw: 5’-CTCGCAACTCTTTACACCGTAGA-3’  Rv: 5’-TTTCCTCCAGCACCTGTAATCTC-3’ |
| SerpinA1 | Fw: 5’-CCGCCATCTTCTTCCTGCCTGA-3’  Rv: 5’-CCGGAGAGGTCAGCCCCATTG-3’ |
| ZO-1 | Fw: 5’-TCTTCAAAGGGAAAGCCTCCTG-3’  Rv: 5’-TTACCTTCACCATGTGCTCCC-3’ |
| OCLN | Fw: 5’-ACAGCAGCGGTGGTAACTTT-3’  Fw: 5’-ATATTCCCTGATCCAGTCCTCCTC-3’ |
| CLDN2 | Fw: 5’-CCCCTTGTACTTCGCTCCCC-3’  Fw: 5’-TAGAAGACAGGGCAGTTCTTTGCA-3’ |
| B-ACT | Fw: 5’-CTTTGCCGATCCGCCGC-3’  Rv: 5’-ATCACGCCCTGGTGCCTGG-3’ |
| CYP24A1 | Fw: 5’-CGCAAATACGACATCCAGGC-3’  Rv: 5’-AATACCACCATCTGAGGCGT-3’ |
| VDR | Fw: 5’-TGCTATGACCTGTGAAGGCTG-3’  Rv: 5’-AGTGGCGTCGGTTGTCCTT-3’ |
| CYP27B1 | Fw: 5’-GTCCAGACAGCACTCCACTC-3’  Rv: 5’-ACCACAGGGTACAGTCTTAGC-3’ |
